# Therapeutic Serendipity Secondary to Abdominal Drain in Pancreatic Trauma

**DOI:** 10.7759/cureus.3870

**Published:** 2019-01-11

**Authors:** Jojo James, Ajay Belgaumkar, Ameet Patel

**Affiliations:** 1 Surgery, Tata Main Hospital, Jamshedpur, IND; 2 Surgery, Kings College Hospital, London, GBR

**Keywords:** pancreatic trauma, fistula, drainage

## Abstract

A case of blunt pancreatic trauma during a soccer match is presented in a young adult female. Following diagnosis, a laparotomy was performed and multiple abdominal drains placed. A controlled pancreatic fistula occurred, which was treated conservatively. Spontaneous drain migration into the duodenum created a therapeutic iatrogenic internal fistula.

## Introduction

Surgical drains have been a vital and crucial step to drain abdominal and thoracic cavity in many surgical procedures.

It is more mandatory following a trauma surgery to drain any expected or unanticipated collection which may subsequently occur after surgery. The surgeon decides on duration of the drain by analysing the contents physically to asses if any leak has happened or biochemically drain output may be analysed for amylase to rule out a pancreatic fistula.

The surgeon is aware that the drains may get blocked by either omentum or clots and that the benefit expected may not be achieved, moreover prolonged drains may be associated with complication like drain migration or erosions.

We wish to report an unusual complication of surgical drains in pancreatic trauma which according to our knowledge is one of the first cases reported which has incidentally benefited our patient.

## Case presentation

**Figure 1:**
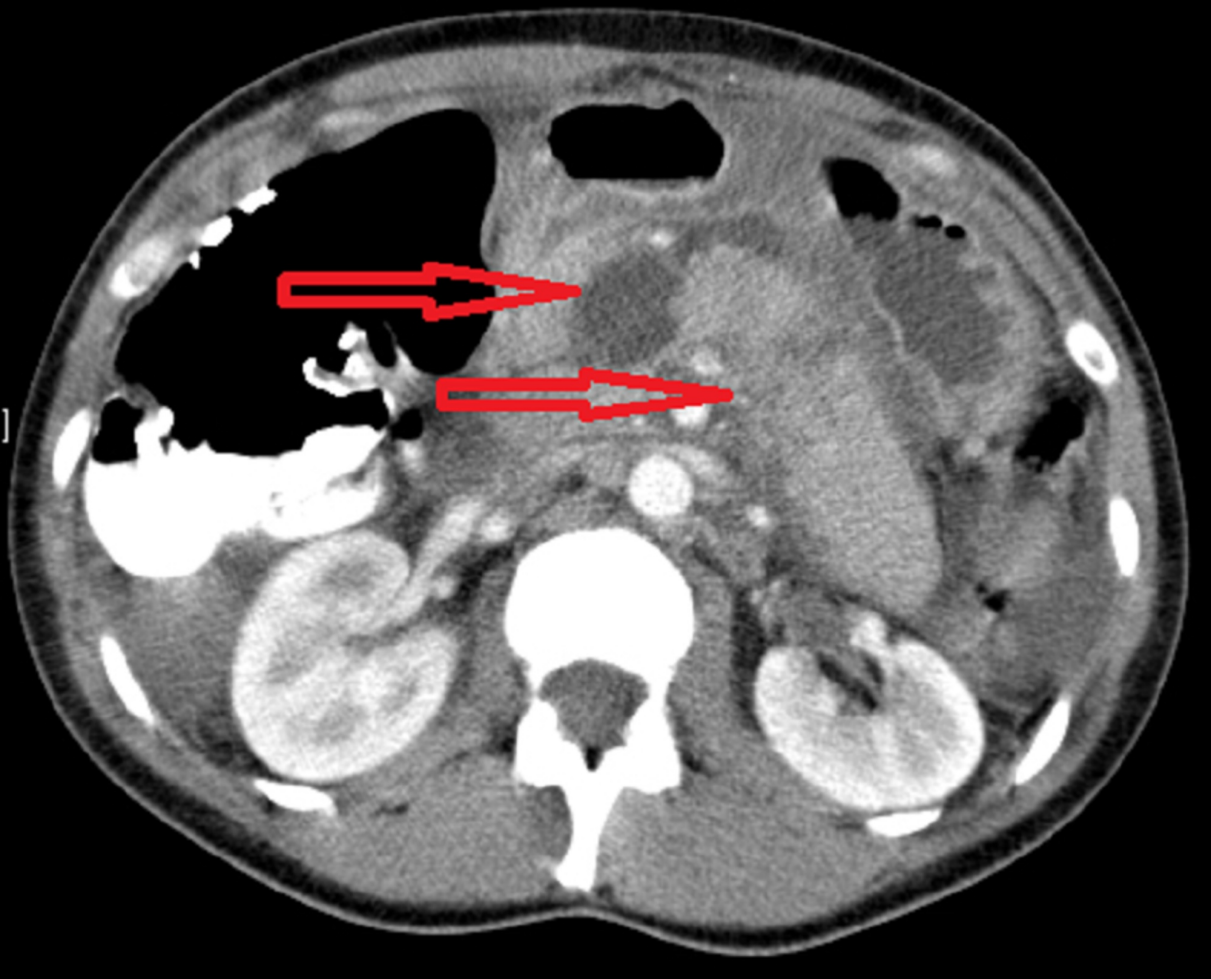
Computed tomography (CT) scan showing significant injury to pancreatic neck and body. Top arrow: neck; bottom arrow: body.

A 32-year-old female goalkeeper developed severe abdominal pain and vomiting after a collision during a soccer game. At presentation, she was haemodynamically stable, with generalised abdominal tenderness and signs of peritoneal irritation. Investigations showed hyperamylasaemia (more than 1000 iu/L), leucocytosis and metabolic acidosis. Computed tomography (CT) demonstrated a major parenchymal injury of the proximal pancreas (Figure 1).

**Figure 2:**
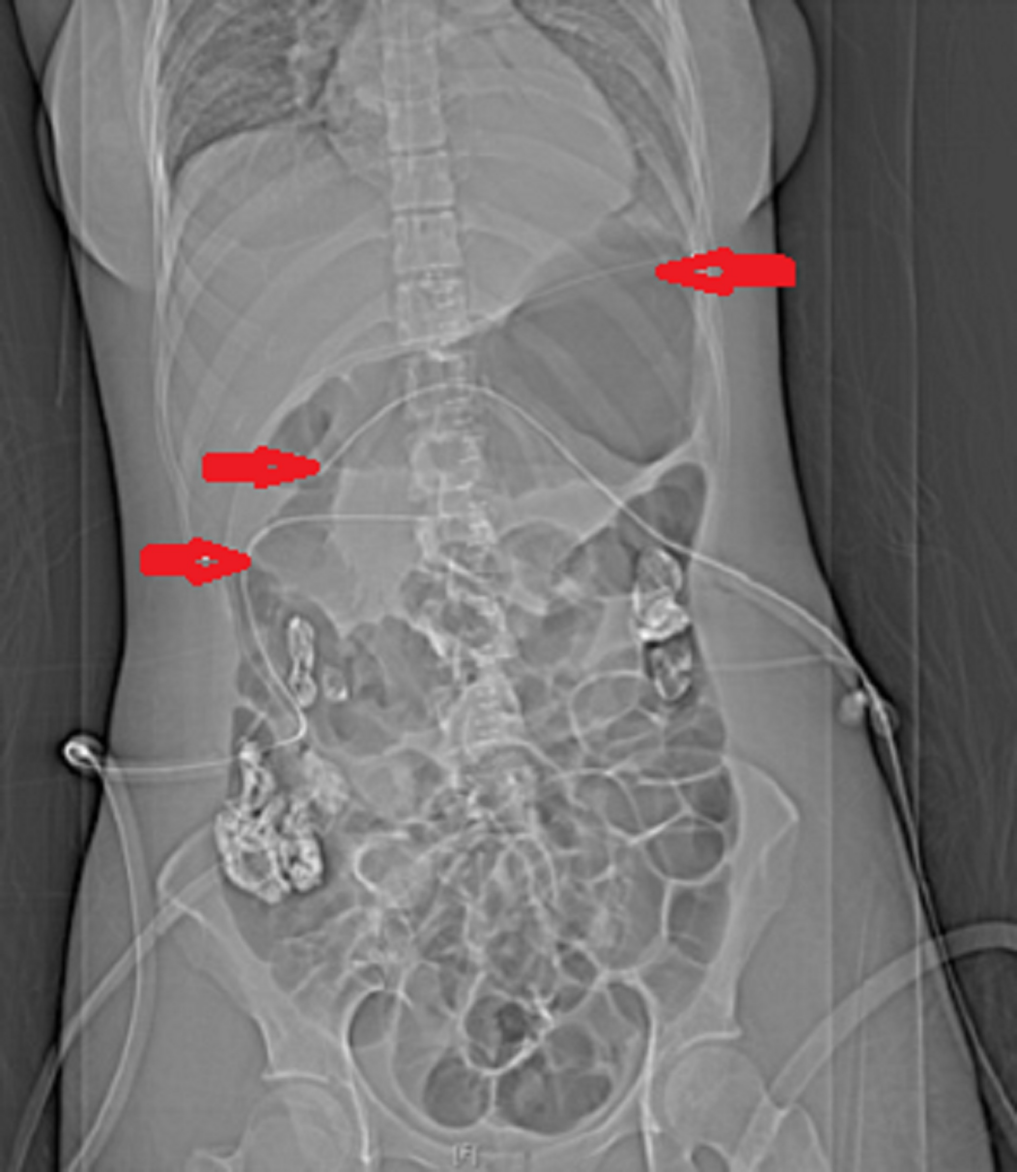
Post-operative abdominal plain X-ray demonstrating position of drains. Arrows show three large closed suction drain sites: Uppermost arrow - adjacent to the pancreas Second arrow - the subhepatic space Third arrow - the subphrenic spaces

She was transferred to a tertiary hepatobiliary centre for further treatment. At laparotomy, transection of the neck of pancreas was seen with a small laceration of the body and no ductal injury. The duodenum was intact and viable. Intra-operative peritoneal fluid amylase was 381 iu/L, compared with serum amylase 311 iu/L, also suggesting no significant pancreatic duct (PD) injury. After thorough lavage, three large closed suction drains were placed adjacent to the pancreas and in the subhepatic and subphrenic spaces (Figure 2).

**Figure 3:**
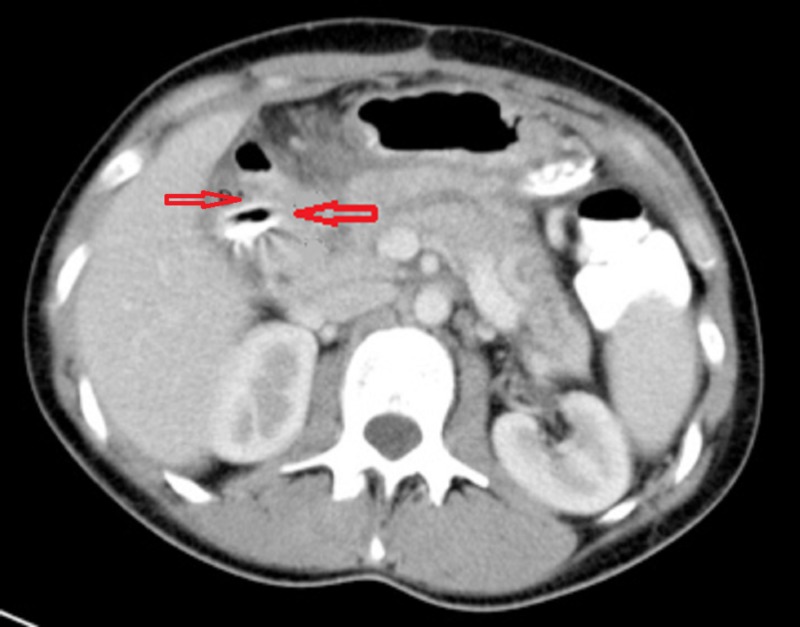
Repeat computed tomography (CT) scan demonstrating migration of lesser sac drain into duodenum. Upper arrow indicates duodenum. Lower arrow shows the path of drain.

On post-operative day one, the drain output was over 500 mls/day and drain fluid amylase was 10484 iu/L, confirming PD leak. This high output pancreatic fistula persisted. The patient was managed conservatively, with parenteral then nasojejunal feeding. Four weeks post-injury, she was suitable for discharge home with the nasojejunal tube and lesser sac drain in situ. An endoscopic retrograde cholangiopancreatography (ERCP) with pancreatic stent insertion was planned in a further four weeks. The patient was reviewed weekly as an outpatient and remained well in the interim period. She was admitted the day before her scheduled ERCP (eight weeks following injury), with sudden onset abdominal pain and bile in the drain, with no signs of generalised peritonitis. CT confirmed that the drain had eroded into the second part of the duodenum (Figure 3).

**Figure 4:**
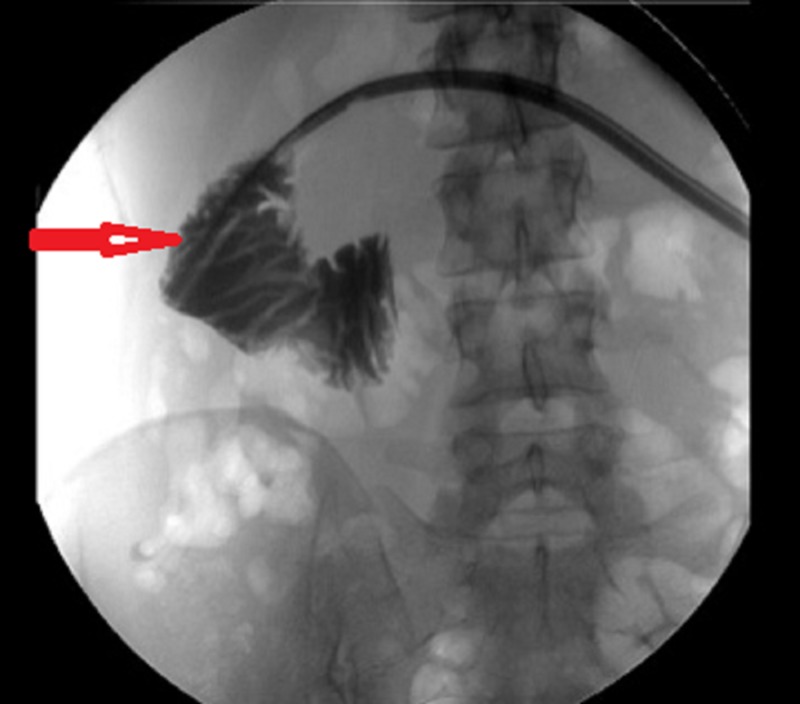
Tubogram showing free flow of contrast through drain into duodenum. Arrow shows duodenum.

Tubogram demonstrated free flow of contrast into the small bowel (Figure 4).

Her abdominal pain settled and she remained well, so the patient was managed conservatively. After three weeks, the drain was removed without complication.

## Discussion

Blunt pancreatic trauma typically occurs in adults following motor vehicle collision and bicycle handlebar injury. PD injury is the primary cause of major complications, including sepsis, intra-abdominal collections and pancreatic fistulae [1]. Duct injuries should be suspected even if initial CT does not show any main PD disruption [2]. At laparotomy, emergency pancreatic resection or a covering Roux loop pancreaticojejunostomy may be considered in high-grade injuries in stable patients. Aggressive drainage of the lesser sac should be performed in all cases, given the high incidence of pancreatic fistulae (up to 40%) [1]. Once a pancreatic fistula is diagnosed, treatment is directed at improving nutritional status, treating sepsis and ensuring adequate drainage of any collections [1]. ERCP with sphincterotomy and PD stenting may facilitate healing of established fistulae by reducing intra-ductal pressures, although there is a long-term risk of duct strictures [3]. Migration of abdominal drains is a feared iatrogenic complication, with a risk of causing bleeding, sepsis and perforation. There are no other reports of drain migration inadvertently leading to a therapeutic pancreatico-duodenal fistula. Three years post-injury, the patient remains asymptomatic and has started playing soccer again.

## Conclusions

With the upsurge in trauma cases presenting to an emergency surgeon the amalgamation of surgical techniques during management of any pancreatic injury during any emergency has increased. Most of the surgeons do prefer to put a drain to evacuate any collection especially main pancreatic duct injury as any collections can cause catastrophic complication like haemorrhage, shock, or even death. The frequency of pancreatic fistula post pancreatic surgery is around 9% and 13%. Hence routine placement of drain is acceptable by a multitude of surgeons to drain any anastomotic leaks, pancreatic fistulas, bleeding, or other intra-abdominal fluid collections. However internal fistulisation of drain effectively solved the postoperative collection of our patient. This article analyses the affirmative effect of routinely placed drain which later migrated into the bowel causing internal fistulisation and drainage.
